# Telomerase Reverse Transcriptase Synergizes with Calorie Restriction to Increase Health Span and Extend Mouse Longevity

**DOI:** 10.1371/journal.pone.0053760

**Published:** 2013-01-22

**Authors:** Elsa Vera, Bruno Bernardes de Jesus, Miguel Foronda, Juana M. Flores, Maria A. Blasco

**Affiliations:** 1 Telomeres and Telomerase Group, Molecular Oncology Program, Spanish National Cancer Research Centre, Melchor Fernández Almagro 3, Madrid, Spain; 2 Animal Surgery and Medicine Department, Facultad de Veterinaria, Universidad Complutense de Madrid, Madrid, Spain; University of Valencia, Spain

## Abstract

Caloric restriction (CR), a reduction of food intake while avoiding malnutrition, can delay the onset of cancer and age-related diseases in several species, including mice. In addition, depending of the genetic background, CR can also increase or decrease mouse longevity. This has highlighted the importance of identifying the molecular pathways that interplay with CR in modulating longevity. Significant lifespan extension in mice has been recently achieved through over-expression of the catalytic subunit of mouse telomerase (mTERT) in a cancer protective background. Given the CR cancer-protective effects in rodents, we set to address here whether CR impacts on telomere length and synergizes with mTERT to extend mouse longevity. CR significantly decreased tumor incidence in TERT transgenic (TgTERT) mice and extended their lifespan compared to wild-type (WT) controls under the same diet, indicating a synergy between TgTERT and CR in increasing mouse longevity. In addition, longitudinal telomere length measurements in peripheral blood leukocytes from individual mice showed that CR resulted in maintenance and/or elongation telomeres in a percentage of WT mice, a situation that mimics telomere dynamics in TgTERT cohorts. These results demonstrate that CR attenuates telomere erosion associated to aging and that synergizes with TERT over-expression in increasing “health span” and extending mouse longevity.

## Introduction

Caloric restriction (CR) in various organisms, including primates, delays the development of some age-related diseases such as cancer, atherosclerosis, diabetes, and neuro-degenerative and respiratory failures, among others [1], [2], [3], thus increasing the so-called “health span”. At the metabolic level, CR results in improved insulin sensitivity and subsequent decrease in the fasting glucose, protecting from age-dependent metabolic syndrome and diabetes [2], [4], [5]. CR in humans also reduces the risk factor for diabetes, cardiovascular disease and cancer [4], although it has been reported to negatively impact on bone mineral density and muscular mass [6], [7].

In addition to these beneficial effects of CR in increasing “health span”, chronic CR is considered among the most robust life-extending interventions, although several recent reports indicate that CR does not always extend and may even shorten lifespan depending on the genotype [2], [8], highlighting the importance of finding the genetic pathways which synergize with CR in extending or shortening lifespan. In the particular case of the C57BL/6 mouse strain used in this study, CR has been reported to produce around a 20% life extension when started in 12 months old mice and 40% when started with 19 months old mice [9], [10], [11], [12]. Similar strategies were employed in younger mice and rats and, although the duration was different from the actual study, some beneficial effects could be observed (for a comprehensive review see [13]).

The exact mechanisms by which CR works are currently debated, although the most widespread theory points to a significant protection from DNA damage due to a reduction of metabolism [14], [15]. Understanding the mechanisms underlying CR is of great importance as this could pinpoint new therapeutic targets for age-associated diseases, or for anti-aging therapies. In this regard, the well-documented association between telomere shortening and aging [16] suggests a possible role of telomere dynamics in the systemic effects of CR.

Telomeres protect chromosome ends from degradation and DNA repair activities and, therefore, are essential for chromosome-end integrity (telomere capping) and chromosomal stability [17]. Telomere repeats are maintained by telomerase, a reverse transcriptase that can elongate chromosome ends *de novo* in those cells where it is expressed at sufficiently high levels, such as embryonic pluripotent stem cells [18]. In telomerase-negative cells, telomeres become shorter associated to each round of cell division due to the end-replication problem and to the action of DNA degrading activities. Short telomeres are passed onto daughter cells and thus telomere shortening is exacerbated with cell division, as well as with increasing age both in humans and mice [19]. Critically short telomeres can trigger a persistent DNA damage response, which leads to cellular senescence and/or apoptosis [20], thus eventually compromising tissue function and tissue regenerative capacity, and contributing to organismal aging [21]. This progressive telomere shortening is proposed to represent a “molecular clock” that underlies organism aging.

Both telomerase-deficient mice and human diseases involving mutations in telomerase components result in accelerated-aging phenotypes probably due to the depletion of the pools of stem cells followed by organ failure [16], [22], [23]. In addition, the speed of telomere shortening with aging can be influenced by factors known to be a risk for disease and premature death, such as psychological stress, smoking, cognitive impairment and obesity [24]. Little is known, however, on the potential effect of treatments that increase lifespan, such as CR, on the rate of telomere shortening with aging.

Besides CR, lifespan extension has been also achieved by over-expressing the catalytic subunit of telomerase, mTERT, in a cancer protective backgrounds owe to increase expression of tumor suppressor genes [25] or through telomerase expression in old mice by using a gene therapy approach [26]. In this context, mTERT over-expression was sufficient to decrease telomere damage with age, delay aging, and increase median longevity. Transgenic overexpression of mTERT, however, was found to increase cancer incidence, therefore masking the potential beneficial effects of constitutive telomerase activation [27]. Since CR is partially mimicking a tumor suppressive condition, we set here to study the impact of transgenic telomerase overexpression in a CR model.

To this end, we performed longitudinal telomere length analyses in single mice by using an automated highthroughput (HT) quantitative telomere FISH platform, HT-QFISH [28], which allows the quantification of individual telomeric spots, and therefore the percentage of short telomeres, in individual cells from large human and mice cohorts. The abundance of critically short telomeres, rather than the mean telomere length, is indicative of telomere dysfunction [29], and thus likely to be useful as biomarker of aging and age-associated diseases.

In summary, we address here the effect of CR on telomere dynamics and telomere function longitudinally during the lifetime of wild-type and telomerase transgenic mice in a C57BL/6 genetic background, as well as study its impact on several health indicators, cancer, and longevity. In this context, we demonstrate that CR slows down telomere shortening and the accumulation of telomere damage with aging in CR WT mice, a situation that mimics mTERT over-expression. These positive effects of CR on telomere length are observed in a wide range of tissues, including peripheral blood mononuclear cells. Importantly, under our experimental settings TgTERT mice under CR show a significant lifespan extension compared to wild-type mice under CR. In contrast, wild-type mice under CR did not present a significant lifespan extension compared to wild-type control mice. These results demonstrate that CR synergizes with telomerase expression resulting in a significant lifespan extension. A similar synergism was previously observed between telomerase expression and higher level of tumor suppressors, which result in a safe cancer protective background for telomerase expression [25]. Hypothetically, the synergism between telomerase expression and caloric restriction could be ruled though the same mechanism.

## Results

### Calorie Restriction Leads to Significant Weight Loss in both WT and TgTERT Mice

**Figure 1:**
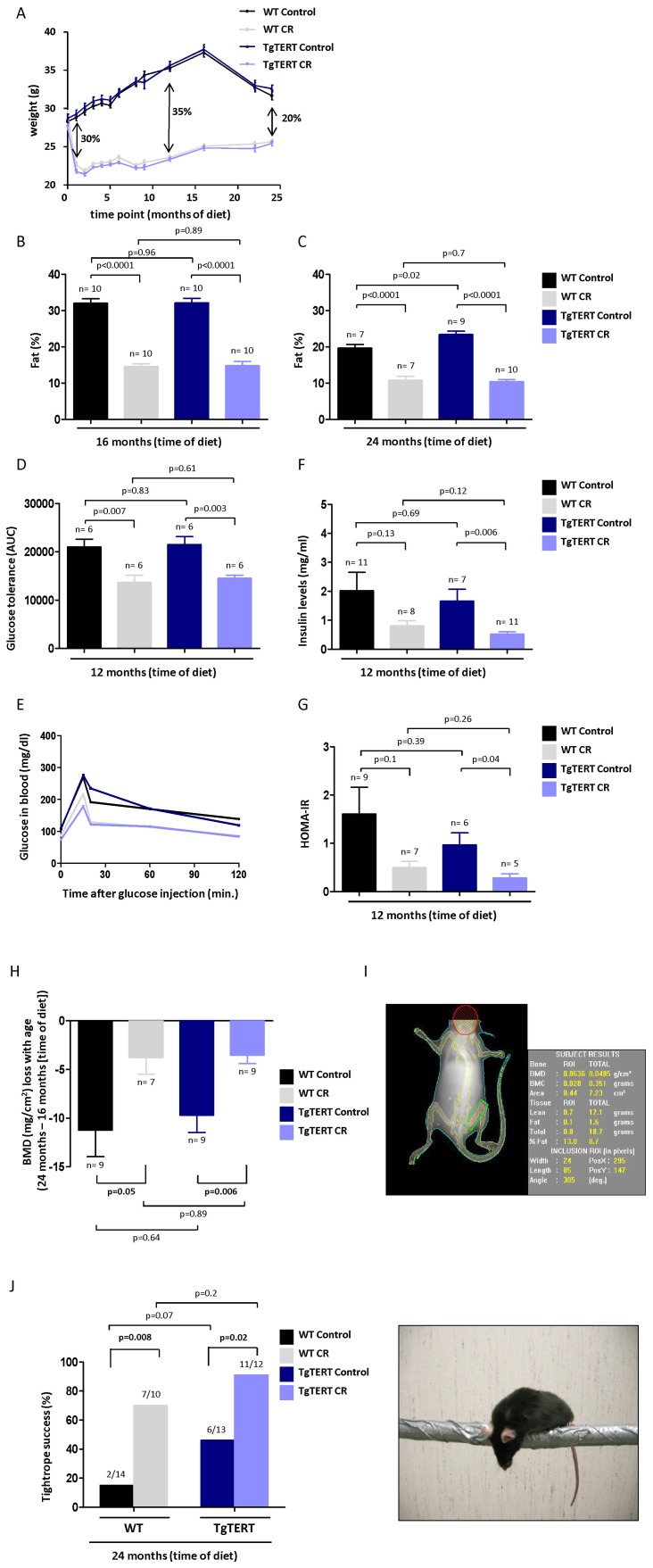
Impact of long-term calorie restriction on metabolic homeostasis and age-associated pathologies. (A) Weight given as average ± SEM of WT and TgTERT mice fed with control or CR diet (see Materials and Methods). One way ANOVA was used to assess statistical significance between the four groups (WT Control vs. Wt CR: p<0.0001; TgTERT Control vs. TgTERT CR: p<0.0001; WT Control vs. TgTERT Control: p = 0.72; WT CR vs. TgTERT CR: p = 0.46). (B and C) Total fat mass of the indicated cohorts was measured at 16 months of diet (B) and 24 months of diet (C). Values are given as average ± SEM, and statistical significance was determined by the two-tailed Student’s t-test. (D and E) Glucose tolerance test (GTT) was performed at 12 months of diet. Integrated AUCs (area under the curve; (D)) and curves (E) are shown. Values are given as average ± SEM, and statistical significance was determined by the two-tailed Student’s t-test. (F) Fasting plasma insulin levels, given as mean ± SEM, was measured in the different cohorts at 12 months of diet. Statistical significance was determined by the two-tailed Student’s t-test. (G) Insulin sensitivity, estimated using the homeostatic model assessment score (HOMA-IR), was performed at 12 months of diet. Values are given as average ± SEM, and statistical significance was determined by the two-tailed Student’s t-test. (H) Femur bone mineral density (BMD) variation through lifetime of WT and TgTERT mice under control and CR diets. Values are given as average ± SEM, and statistical significance was determined by the two-tailed Student’s *t*-test. (I) Representative DEXA image used for BMD and fat mass calculations. (J) Neuromuscular coordination was quantified as the percentage of mice that pass with success the tightrope test. Numbers above the bars represent the number of mice that successfully pass the test over the total number of mice tested. Student’s *t*-test was used to assess significance between control and CR mice. Values are given as average ± SEM, and statistical significance was determined by the two-tailed Student’s *t*-test.

We first set to address whether CR impacts on telomere length dynamics in mice. To this end, we established 4 cohorts of mice, including wild-type and TgTERT mice under either a control or a CR diet. All mice had an identical genetic background, which was a pure C57BL/6 (Materials and Methods). When mice were 3 months of age, those mice to be under CR where shifted to a diet containing 40% of the calories of those of the control diet group. Of note, control mice were not fed *ad libitum*, thus avoiding individual variations in caloric intake in this group (Materials and Methods). One month after the start of diets, the body weight of WT and TgTERT mice under CR was significantly lower than that of the corresponding cohorts under the control diet, and we did not detect differences associated to telomerase over-expression **(** **Fig. 1A**; p<0.0001**)**. After 12 months of diet, both wild-type and TgTERT mice under CR were 35% lighter than the corresponding controls (**Fig. 1A**) and this difference was reduced to 20% at 24 months of diet **(** **Fig. 1A**; p<0.0001**)**. Total fat mass values as determined by DEXA (Materials and Methods), were significantly reduced following 16 months of CR diet in both genotypes compared to the corresponding cohorts under the control diet **(** **Fig. 1B,C** **)**. Those differences were maintained after 24 months of diet, at which point mice under the control diet also presented reduced fat most likely owe to the normal aging process **(** **Fig. 1B,C** **)**. Interestingly, old TgTERT mice under the control diet showed significantly higher total fat mass than the age-matched WT cohort under the same diet **(** **Fig. 1C**; p = 0.02**)**, in accordance with our previous observations that TERT over-expression leads to improved health status and delayed aging associated pathologies [**25**].

### Stronger Effect of CR in Delaying Age-associated Pathologies in Mice Over-expressing TERT

To test the potential synergistic effects of CR and TgTERT in protecting from age-associated pathologies, we first examined the metabolic capacity of mice under CR compared to mice under the control diet. Glucose intolerance and insulin resistance are common indicators of aging in control conditions. In turn, enhanced glucose tolerance and insulin sensitivity normally accompany lifespan extension in mammals and are also observed after long-term CR [7], [30] as well as in some long-lived genetically modified mice, such as mice with enhanced expression of TERT or with enhanced expression of certain tumor suppressor genes [25]. As expected, after 12 months of diet (at 15 months of age), both WT and TgTERT mice under CR showed a significantly improved glucose tolerance compared to the corresponding cohorts under the control diet **(** **Fig. 1D,E**; p = 0.007 and p = 0.003, respectively**)**, as indicated by a faster glucose uptake following glucose injection in fasting (Materials and Methods). Fasting plasma insulin levels **(** **Fig. 1F** **)** showed a trend to be lower in CR WT mice, and this trend reached statistical significance in TgTERT mice after 12 months of CR diet compared to the corresponding cohorts under a control diet **(** **Fig. 1F**; p = 0.006**)**. Similarly, insulin sensitivity after 12 months of treatment tended to be lower in CR WT, and this trend became significant only in CR TgTERT mice **(** **Fig 1G**; p = 0.04**)**, as assessed using the homeostatic model assessment score (HOMA) [**31**].

Another common feature of aging is the development of osteoporosis, a process were bone mineral density (BMD) is reduced [32]. In line with this, both WT and TgTERT mice showed BMD loss through life, as measured by DEXA, however, bone loss was significantly higher in mice under the control diet compared to calorie restricted mice **(** **Fig 1H**; p = 0.05 and p = 0.006 for WT and TgTERT, respectively; **Fig. 1I** for representative image**)**. Of note, in the first 16 months of diet, CR led to decreased BMD in both genotypes compared to the control diet, in agreement with previous observations **(Fig. S1A–C)** [6], [7]. In summary, these findings suggest a long-term protective effect of CR in the onset of osteoporosis in both genotypes in spite of an initial negative impact of CR on bone density.

An additional biomarker of aging is the progressive loss of neuromuscular coordination that can be measured by the tightrope success test [33]. In this assay, CR mice from both genotypes performed significantly better than mice under the control diet **(** **Fig. 1J**; p = 0.008 and 0.02 for WT and TgTERT mice, respectively**)**. Of notice, TgTERT mice under the control diet performed better than WT mice under the same diet, although the differences did not reach statistical significance **(** **Fig. 1J**; p = 0.08**)**, in line with an improved health status and delayed aging associated to TERT over-expression [25]. In summary, CR has a measurable positive effect on neuromuscular coordination in WT mice and this effect seems to be synergistic with that of TgTERT expression.

Together, these results demonstrate that the long-term calorie restriction protocol performed here delays the onset of age-associated pathologies such as glucose intolerance, osteoporosis, and impaired neuromuscular coordination both in WT and TgTERT mice, although in some assays the effects tended to be of a greater magnitude in the TgTERT cohorts (i.e. insulin levels or tightrope test).

### The Impact of CR Decreasing Molecular Markers of Aging is More Apparent in Mice Over-expressing TERT

Calorie restriction, similarly to the effect of some longevity-enhancing mutations in mice, has been shown to reduce IGF1 serum levels, particularly in aged rodents [34], [35]. In our study, however, we did not find significant differences in IGF-1 levels between WT mice under either CR or a control diet **(Fig. S1D**; p = 0.2**)**. Interestingly, IGF-1 levels where higher in TgTERT mice compared WT mice under control diet (**Fig. S1D**; p = 0.01**)**, in agreement with previous findings [25], and these levels were significantly reduced in TgTERT cohorts under CR **(Fig. S1D**; p = 0.04**)**. Growth hormone (GH) levels were significantly increased in TgTERT mice under CR **(Fig. S1E**; p = 0.04**)** compared to the control diet cohorts.

An additional molecular marker of aging is the accumulation of phosphorylated histone H2AX (γ-H2AX) foci in aged tissues, which has been shown to co-localize with double strand breaks (DSBs) as well as with critically short/dysfunctional telomeres [36], [37], [38], [39], [40], [41]. We found a tendency to have decreased levels of γ-H2AX in the kidney of WT and TgTERT CR mice compared to those cohorts under control diet, although the differences were not significant **(Fig. S2A,B)**. The accumulation of γ-H2AX was significantly attenuated, however, only in CR TgTERT mice compared to CR WT cohorts **(Fig. S2A,B**; p = 0.03**)**, suggesting that TERT and CR may contribute in a synergic way to prevent DNA damage with aging something not surprising, since telomerase over-expression prevents telomeres shortening [26], [42] (a form of DNA damage [41]) and CR has been extensively linked to DNA damage protection [43].

### Calorie Restriction Decreases the Rate of Telomere Shortening with Aging in Longitudinal Studies

**Figure 2:**
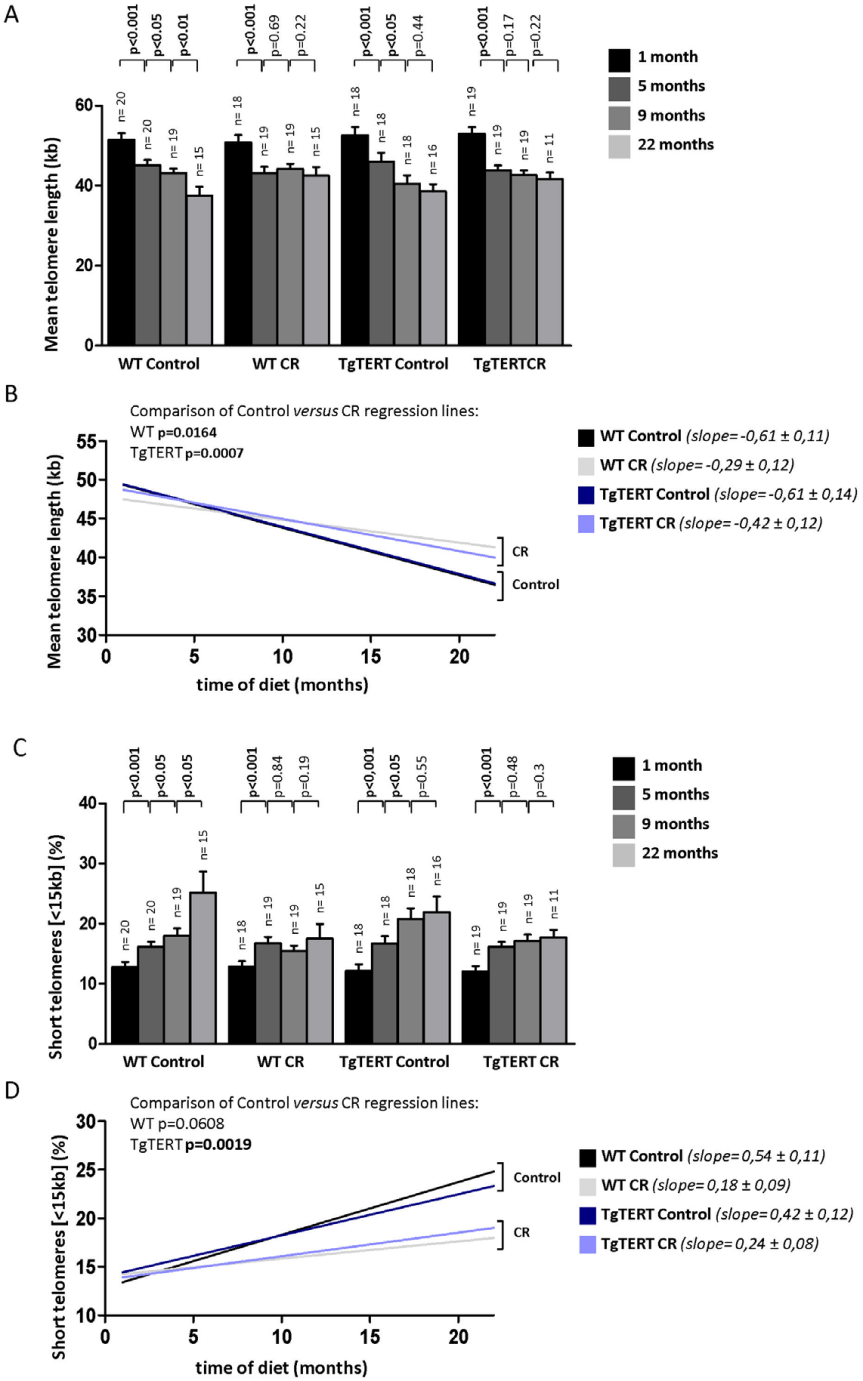
Slower age-dependent telomere shortening in mice under calorie restriction. (A and C) Mean telomere length (A) and percentage of short telomeres (C) was determined by HT QFISH on white blood cells from the indicated mice under CR and control diet. The number of mice is indicated on the top of each bar (n). Values are given as average ± SEM, and statistical significance was determined by one-tailed Student’s *t*-test. (B and D) Linear regression lines of the values obtained for mean telomere length (B) and percentage of short telomeres (D) measured in white blood cells. The slope ± SD of each regression line is indicated and represents the rate of telomere loss with time.

To address the effect of CR in telomere length dynamics, we performed a longitudinal telomere length study in individual WT and TgTERT mice under calorie restriction or under a control diet. The most widely used cell type for human and mouse telomere studies are peripheral blood leukocytes (PBL). Telomere length in PBLs has been proposed to reflect on the speed of aging process as hematopoietic stems cells proliferate throughout life. To this end, first, we measured telomere length and the percentage of short telomeres (arbitrarily set to <15 kb as this cutoff has been previously shown by us to be indicative of presence of short telomeres in mice [26]; see also **Fig. S3A–C** for additional cutoffs) in PBLs by using the highthroughput QFISH (HT-QFISH) method previously developed by us for blood samples (Materials and Methods) [28]. Blood was extracted from each individual mouse after 1, 5, 9 and 22 months of diet. We represented changes in mean telomere length and the percentage of short telomeres (<15 Kb) with time. As recently reported by us, WT mice under the control diet showed significant telomere attrition with age, which was detectable when comparing 4 months intervals, as reflected both by a significant decrease in mean telomere length and by a significant increase in the percentage of short telomeres **(** **Fig. 2A,C** **and Fig. S3A–C)**, demonstrating that mice undergo telomere shortening associated to the aging process [44]. A similar trend was observed for TgTERT under a control diet, however, in this background telomeres shortened only during the first 9 months of diet, after which telomere erosion rate as well as the rate of increase of short telomeres were decreased, most likely as the consequence of increased TERT expression (Fig. 2A,C). Interestingly, in both CR WT and CR TgTERT mice, after an initial telomere shortening during 1 to 5 months of diet, telomeres were stabilized and did not show significant shortening until the end of the experiment **(** **Fig. 2A** **)**. A similar trend was observed for the percentage of short telomeres, which showed an initial increase at 5 month of diet but was stabilized from 5 to 22 month of diet **(** **Fig. 2C** **and Fig. S3C)**. These results are also illustrated by the linear regression of telomere length with time of the different groups. In particular, the rate of telomere shortening, as represented by the slope of the regression line, was significantly slower in CR mice of both genotypes compared to the corresponding cohorts under the control diet **(** **Fig. 2B**; p = 0.0164 and p = 0.0007 for WT and TgTERT, respectively**)**. Similarly, WT and TgTERT mice under CR showed a lower rate of accumulation of short telomeres compared to the corresponding cohorts under the control diet **(** **Fig. 2D**; p = 0.0608 and p = 0.0019 for WT and TgTERT, respectively**)**. These results further reinforce our findings that long-term CR decreases the rate of telomere shortening associated with aging, and protects form the appearance of short telomeres. This links two major cellular pathways involved in the aging process demonstrating for the first time that caloric restriction impacts on telomere dynamics.

### Calorie Restriction Leads to Maintenance and/or Elongation of Telomeres in Individual Mice

**Figure 3:**
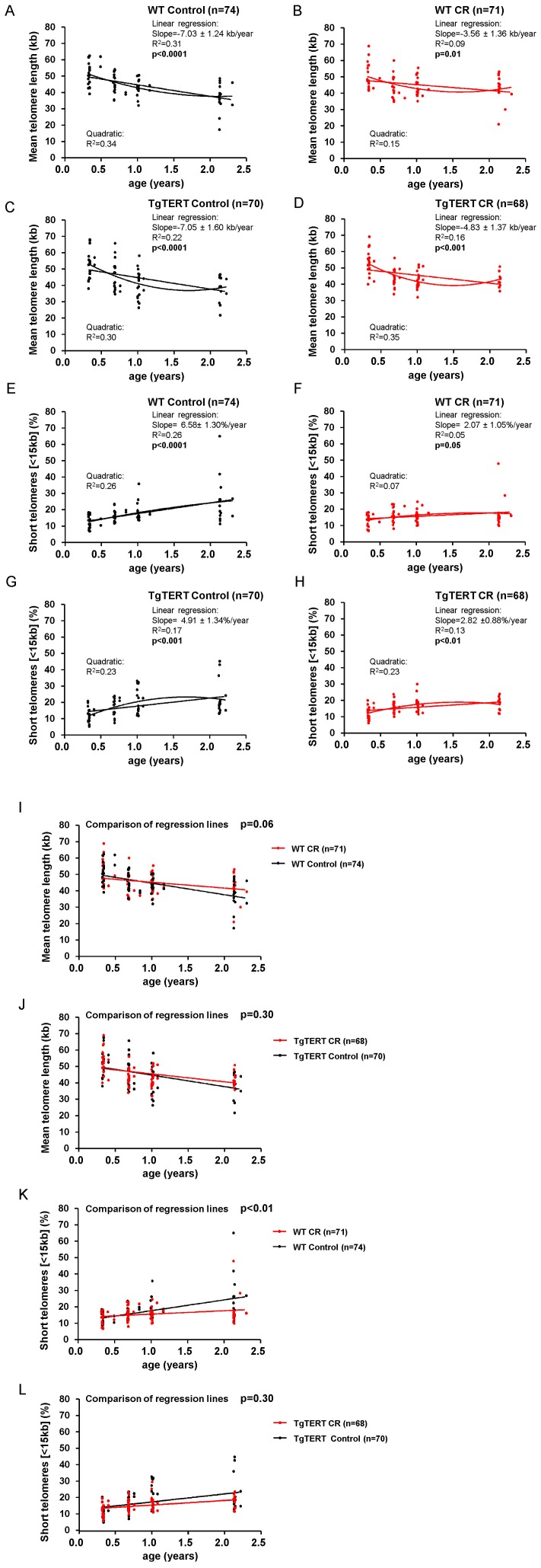
Longitudinal telomere length analyses. (A, B, C and D) Adjustment of mean telomere length values with aging to a linear model or, alternatively, to a quadratic model in the indicated mouse cohorts. Linear regression analysis was used to measure the association between age and mean telomere length. The slope of the regression line is indicated and represents the rate of telomere shortening per year (Kb). Second order polynomial adjustment (quadratic) was used for the non-linear fit model. The R^2^ indicates the goodness of the data adjustment to each model. The number of mice per group is shown (n). (E, F, G and H) Adjustment of the percentage of short telomeres (<15kb) to a linear model or alternatively to a quadratic model in the indicated mouse cohorts. Linear regression analysis was used to measure the association between age and the percentage of short telomeres. The slope of the regression line is indicated and represents the percentage of short telomeres enrichment per year. Second order polynomial adjustment (quadratic) was used for the non-linear fit model. The R^2^ indicates the goodness of the data adjustment to each model. The number of mice per group is shown (n). (I and J) Linear regression lines of the association between age and mean telomere length are shown for mice under CR (red lines) or a control diet (black lines), in both WT (I) and TgTERT (J) backgrounds. Multiple regression analysis was used to evaluate the statistical differences between the slopes of the different linear regression lines. The number of mice in each group is indicated (n). (K and L) Linear regression lines of the association between age and the percentage of short telomeres (<15 kb) are shown for mice under CR (red lines) or a control diet (black lines), in both WT (K) and TgTERT (L) backgrounds. Multiple regression analysis was used to assess the statistical differences between the different linear regression lines. The number of mice in each group is indicated (n).

To understand how CR is impacting on telomere length, we studied telomere dynamics with time in individual mice of both genotypes. First, we plotted both mean telomere values and the percentage of short telomeres at different time points for each mouse and adjusted these values to either a linear model (linear regression) or a non-linear model (quadratic) **(** **Fig. 3A–L** **)**. In a recent report, we described that the rate of telomere shortening per year in both WT mice and TgTERT mice under a control diet is 100-times faster than in humans [44]. Interestingly, here we first show that both the rate of telomere shortening and the rate of accumulation of short telomeres were affected by CR in WT mice (p-value = 0.06 for mean telomere length and p-value<0.01 for the percentage of short telomeres), but not in the TgTERT group **(** **Fig. 3I–L** **)**. These findings indicate that the effects of CR on telomere maintenance are smaller in the telomerase over-expressing mice, suggesting that they could be partially mediated by TERT.

**Figure 4:**
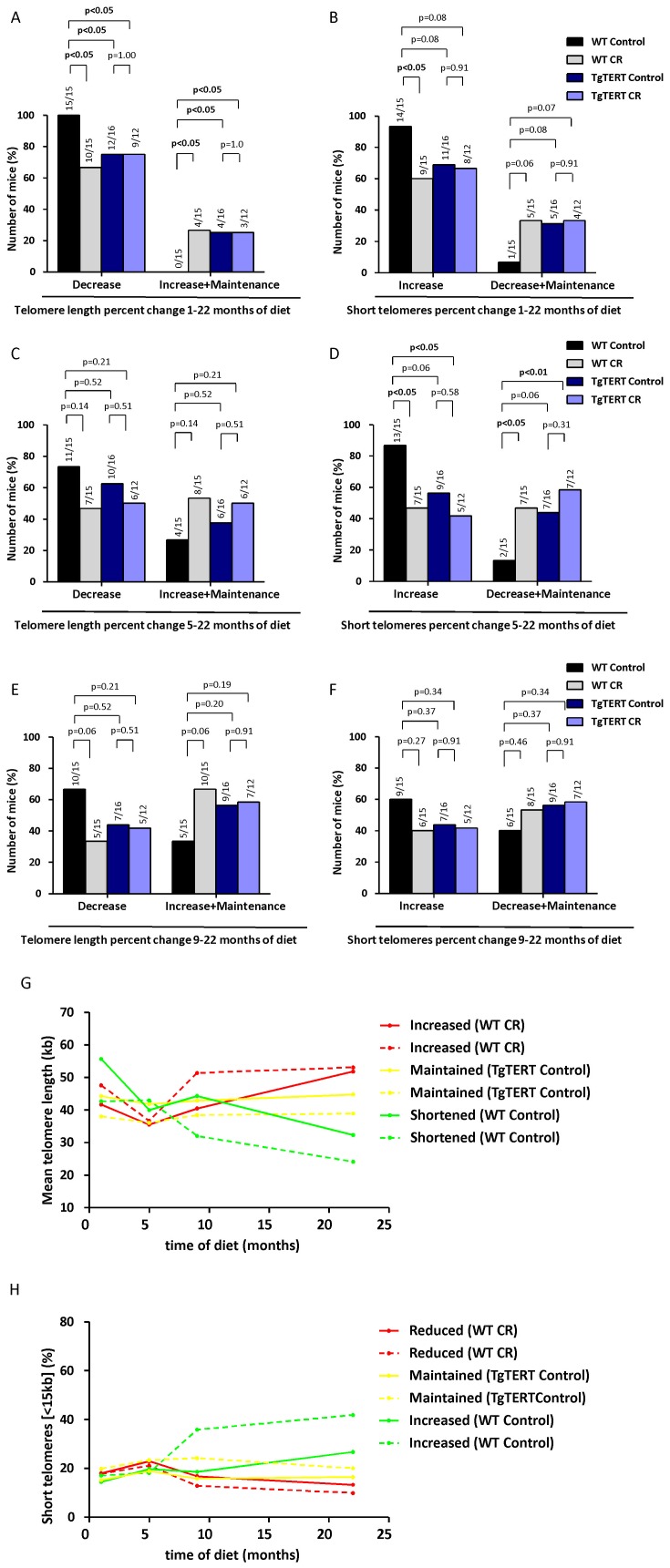
Calorie restriction leads to telomere maintenance and/or elongation with time in a percentage of mice. (A, C and E) The behavior of mean telomere length was classified in two different profiles (“Decrease” and “Increase or Maintenance”) at different times of diet (1–22 months of diet, 5–22 months of diet and 9–22 months of diet; A, C and E, respectively) in the indicated groups. Numbers above bars indicate the number of mice showing the profile of interest over the total number of mice. Chi-squared test was used to assess the statistical significance of the differences observed. (B, D and F) The behavior in the percentage of short telomeres (<15 kb) was classified in two different profiles (“Increase” and “Decrease or Maintenance”) at different times of diet (1–22 months of diet, 5–22 months of diet and 9–22 months of diet; B, D and F respectively) in the indicated groups. Numbers above bars indicate the number of mice with the profile of interest over the total number of mice. Chi-squared test was used to assess the statistical significance of the differences observed. (G and H) Representative examples of the different assigned profiles for mean telomere length (“Increased”, “Maintained”, and “Shortened”) and percentage of short (<15 kb) telomeres (“Reduced”, “Maintained”, and “Increased”).

To address this, we studied different patterns of telomere length behavior per individual mice with time **(** **Fig. 4A–F** **)**. Interestingly, we observed three different trends for mean telomere length behavior: shortening of telomeres, maintenance of telomeres, and elongation of telomeres. Similarly, for the percentage of short telomeres, we found increased, decrease or maintenance in the percentage of short telomeres. These different behaviors are consistent with the fact that leukocytes can activate telomerase [45]. The frequency of these patterns in different genotype mice under CR or control diet may be informative on the effects of CR or TERT over-expression on telomere length dynamics with aging. To this end, we studied the changes in telomere length in 3 time intervals: 1–22, 5–22 and 9–22 months of diet **(** **Fig. 4A–F** **and Fig. S4A–F)**. When considering behavior of telomeres in individual mice during 1–22 months of diet period, all WT mice under the control diet showed telomere shortening with time and none of them showed maintenance or elongation of telomeres during this period. In contrast, both WT mice under CR and TgTERT mice under both diets showed a number of individuals (∼20%) that either elongated or maintained telomere length. This was paralleled by the abundance of short telomeres. Representative examples of each pattern are represented **(** **Fig. 4G,H** **)**. These findings indicate that CR can stop or reverse the trend of continuous telomere shortening with aging, in an analogous manner to that observed for TERT over-expression.

### Calorie Restriction Results in Longer Telomeres in Various Adult Mouse Tissues

**Figure 5:**
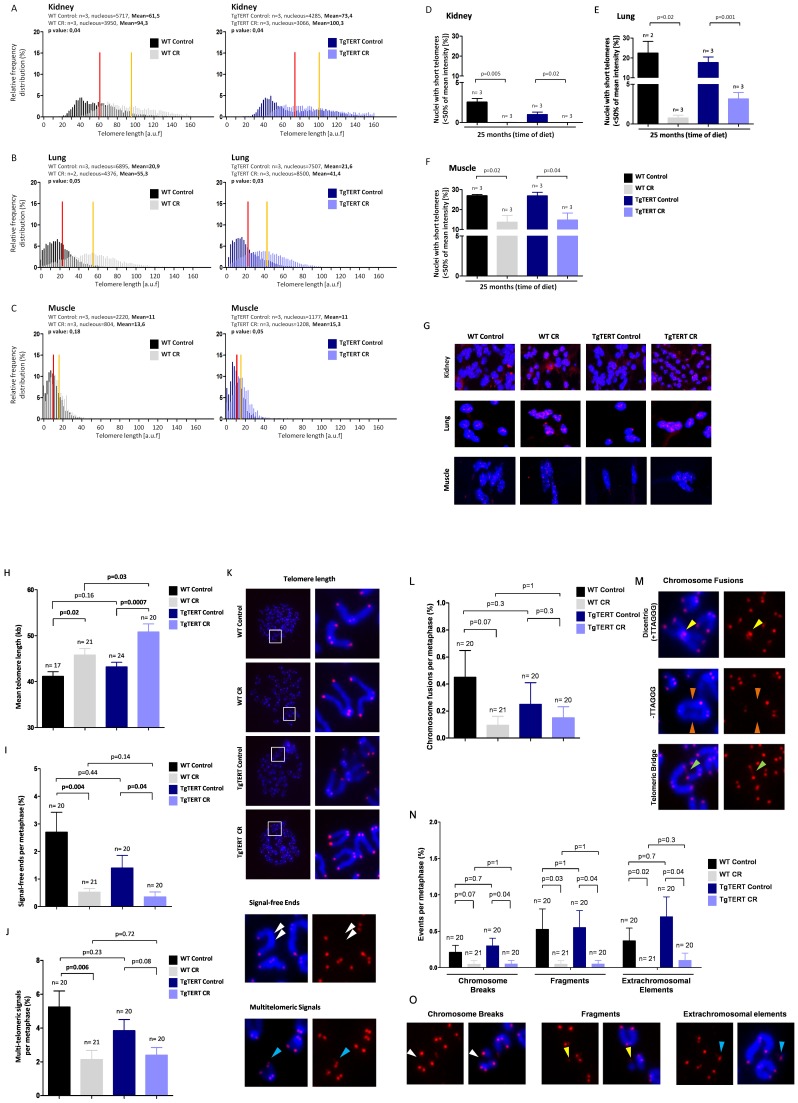
Calorie restriction prevents telomere shortening in different mouse tissues and protects from telomere-mediated chromosomal aberrations in bone marrow cells. (A,B,C) Telomere fluorescence as determined by QFISH in the indicated tissues from the different mouse cohorts studied here. Histograms represent the frequency (in percentage) of telomere fluorescence per nucleus (in arbitrary units of fluorescence [auf]). Mean telomere length is indicated by a straight line, red for mice under a control diet and yellow for mice under CR. The number of mice (n) and the total number of nuclei analysed is indicated. (D,E,F) Percentage of short telomeres (fraction of telomeres presenting intensity below 50% of the mean intensity) in the indicated tissues from the different mice cohorts studied as determined by QFISH. Student´s *t*-test was used for statistical analysis. (G) Representative QFISH images for different tissues from the indicated cohorts. Blue colour corresponds to chromosome DNA stained with DAPI; red dots correspond to telomeres (TTAGGG repeats). (H) Telomere length measured in metaphase spreads of BM cells from the different cohorts after hybridization with DAPI and a fluorescent Cy3 labelled PNA-telomeric probe. (I) Frequency of signal-free ends per metaphase in BM cells from the indicated mouse cohorts. (J) Frequency of multitelomeric signals (MTS) per metaphase in BM cells from the indicated mouse cohorts. (K) Representative QFISH images of metaphases from the indicated mouse cohorts. Blue, DNA stained with DAPI; red, telomeres (TTAGGG repeats). (L) Frequency of chromosome fusions per metaphase in BM cells from the indicated mouse cohorts. (M) Representative images of chromosomal fusions. (N) Frequency of other chromosome aberrations (chromosome breaks, fragments and extrachromosomal elements) per metaphase in BM cells from the indicated mouse cohorts. (O) Representative images of the different types of chromosomal aberrations scored. Blue, DAPI staining (DNA); red dots, telomeres (TTAGGG repeats) as detected with a PNA-Cy3 probe.

To further study the impact of CR in telomere maintenance in the context of the organism, we measured telomere length in a variety of tissues (lung, kidney-cortex, muscle-fibers) from WT and TgTERT mice that had been under CR or a control diet during a total of 23 months. To this end, we used a quantitative telomere FISH method (QFISH) on mouse tissue sections (Materials and Methods). In agreement with the results obtained in leukocytes, tissues from CR mice showed significantly longer telomeres than those of mice under a control diet, as reflected both by higher mean telomere length and by a lower percentage of nuclei with short telomeres **(** **Fig. 5A–H** and representative images in **Fig. 5K** **)**. These results were confirmed by quantitative QFISH on metaphasic chromosomes from bone marrow (BM) cells, which allows measuring all individual telomeres per nuclei. In particular, mice under CR showed longer telomeres and a lower percentage of chromosome ends with undetectable telomere signals by QFISH (or signal-free ends) compared to the corresponding genotypes under a control diet **(** **Fig. 5H,I** **)**. Together, these results indicate a systemic effect of CR on telomere length maintenance in multiple organs in mice.

### Calorie Restriction Decreases Accumulation of Telomere-originated Chromosomal Aberrations with Aging

Telomeres protect chromosome ends from recombination and DNA repair activities, thus preventing end-to-end fusions and other telomere-related chromosomal aberrations [46], [47]. Telomere shortening below a critical length has been shown to lead to loss of telomere protection, chromosomal instability, as well as severe cellular defects (senescence/apoptosis), which eventually can contribute to development of cancer and/or aging.

Here, we used telomere QFISH on BM metaphases to determine the impact of CR on telomere-originated chromosomal aberrations. After 23 months of dietary restriction, CR mice of both genotypes presented a tendency to have less telomere fusions and chromosomes with multitelomeric signals, a telomere aberration recently related to telomere fragility [48], as well as other types of aberrations such as chromosome breaks and fragments **(** **Fig. 5J–O**; representative images at **Fig. 5M,O**).

Altogether, these results indicate that long-term calorie restriction results in protection from telomere damage and occurrence of telomere-originated chromosomal aberrations.

### CR Synergizes with TERT Over-expression in Extending Mouse Longevity

**Figure 6:**
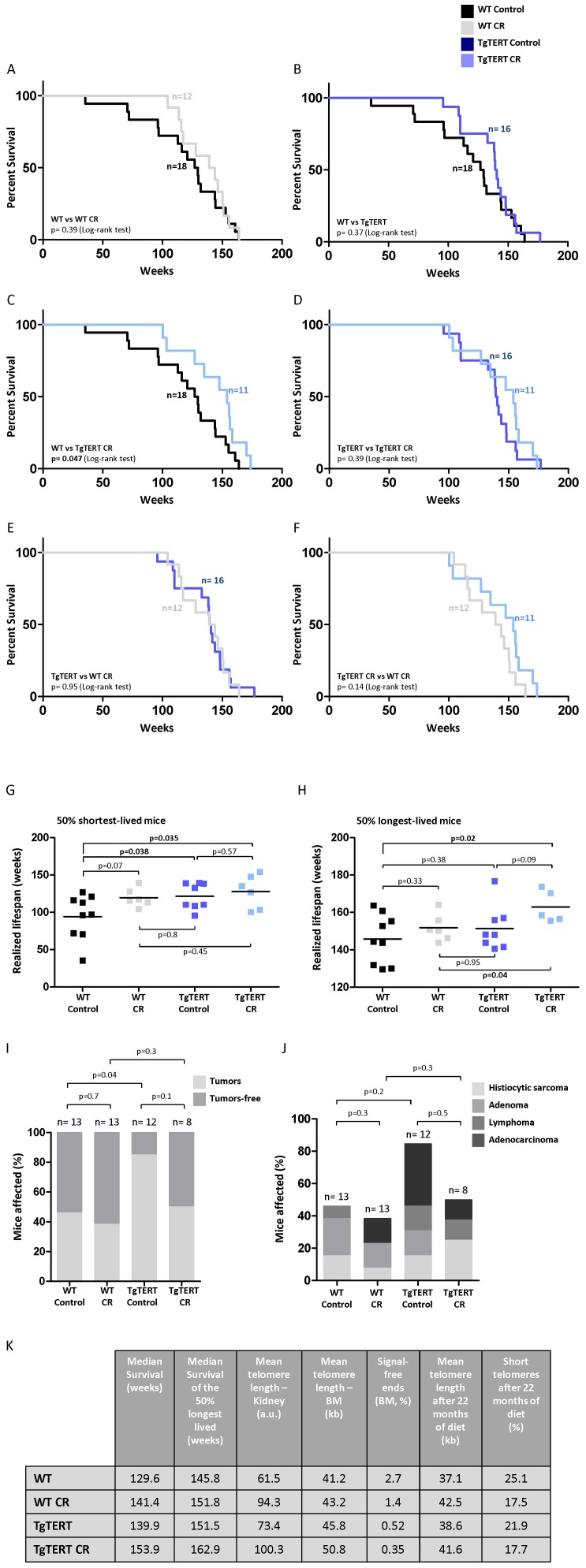
Caloric restriction increases median and maximum longevity and protects from cancer. (A–F) Kaplan-Meyer survival curves of the indicated mouse cohorts. The Log rank test was used for statistical analysis. Mice under CR were more susceptible to unexpected stresses such as the blood extraction procedure carried. 5 mice of the WT CR cohort and 6 mice of the TgTERT cohort died during the blood extraction and were excluded from the survival curves (of note, none of the WT or TgTERT mice show sensibility to the blood extraction procedure). (G–H) Realized lifespan of the 50% shortest (G) and longest (H) lived mice of each cohort. Student´s *t*-test was used for statistical analysis. (I) Percentage of mice with cancer and cancer-free mice in the different cohorts. All death mice were subjected to full histopathological analysis. (J) Percentage of mice in the different cohorts with the indicated tumours at their time of death. (K) Summary table with the findings regarding telomere length and survival of the different cohorts.

Finally, we tested the ability of a CR diet to extend the lifespan of both WT and TgTERT cohorts. We observed a tendency to increase the median survival of WT mice under CR compared to those under a control diet (141, 4 weeks compared to 129,6 weeks, respectively; **Fig. 6A–H**), although the differences did not reach significance (**Fig. 6A**). A possible explanation for the lack of lifespan extension associated to CR in the WT cohort could be related to the early age of the mice at the start of the CR protocol, which was of only 3 months. Nevertheless, we observed that WT mice under CR showed a delay in the time of onset of the first deaths in the colony (**Fig. 6A and I–J**) in agreement with an increased “health span” as the result of CR (see also Figs. 1 and 2). Of note, this effect was similar to that seen in TgTERT mice under a control diet, which also showed an increased median survival of 139, 9 weeks compared to 129,6 weeks in the WT controls, as well as a delayed onset of first deaths in the colony (**Fig. 6B–J**), suggesting that the beneficial effects of CR in increasing “health span” in WT mice could be mediated at least in part by TERT over-expression. Interestingly, we observed a significant extension of the median longevity in the TgTERT mice under CR compared to WT mice under a control diet (**Fig. 6C**). These results suggest a synergistic effect between TERT over-expression and CR in increasing mouse longevity (which could be also observed in **Fig 6H**).

In this regard, we have previously demonstrated a synergistic effect between TERT over-expression and increased expression of tumor suppressor genes [25]. As CR has been also previously demonstrated to protect from different cancer types [5], [12], we next set to address whether CR was significantly impacting on tumor formation in WT and TgTERT cohorts. To this end, we performed full histopathological analysis of all mice at their time of death. Interestingly, both WT and TgTERT cohorts under CR showed a lower incidence of tumors compared to the corresponding cohorts under the control diet **(** **Fig. 6I,J** **)**. Of note, the tumor spectrum associated to TERT over-expression was different from that of control mice **(** **Fig. 6J** **)**, irrespective of this fact, the incidence of cancer in TgTERT mice was reduced to similar levels of those of wild-type mice under a control diet. In summary, CR is able to reduce the increased cancer incidence associated to TERT over-expression, which together with the increased “health span” associated to TERT over-expression could explain the synergistic effects of TERT and CR in increasing longevity **(**see summary table, **Fig. 6K** **)**.

## Discussion

Amelioration of health span, usually alongside with a significant extension of lifespan, has been achieved in the last decades in various species through interventions in major cellular pathways. One of the oldest observations arise from the seminal studies of McCay et al., which reported that rats under a restricted intake of calories showed and extended longevity [49], and that since then has been extended to other animal models [3]. However, more recently, it has become apparent that effects of CR in extending longevity are not universal [2]. Indeed, depending on the genetic background CR can either extend or decrease longevity, highlighting the need of identifying the pathways that interplay with CR. In line with this notion, the molecular mechanisms responsible for lifespan extension through CR are still debated. As an example, mammalian *Sirt1* (yeast protein Sir2), which encodes a NAD-dependent deacetylase and was long believed to mediate life extension associated to calorie restriction in different species, such as *C. elegans* and *Drosophila*, [50], [51], has been recently challenged [52]. Currently, the most widely accepted model is that CR protects from DNA damage through a decrease of metabolism and mitochondrial activity, and this result in a tissue-protective phenotype [4], [53]. Here, we addressed whether CR could impact on telomere dynamics, one of the best-known molecular mechanisms leading to accumulation of DNA damage with aging. In particular, we set to address whether CR may impact on the rate of telomere shortening as well as on the incidence of telomere-originated aberrations associated with aging. Moreover, we set to address whether CR could synergyze with telomerase over-expression in extending life span.

First, we showed that the CR protocol used here was able to protect from the development of pathologies associated with aging in both WT and TgTERT mice, including insulin sensitivity and glucose intolerance, as well as protection from bone loss over time. In addition to protection from age-related pathologies, CR improved other aspects of mouse health such neuromuscular coordination in both genotypes. Together, these results indicate that the CR protocol used in this study was able to increase the “health span” of both WT and TgTERT mice.

In agreement with this, we observed a delayed onset of first deaths in the WT and TgTERT cohorts under CR. Interestingly, WT mice under CR showed a similar median longevity and similar onset of first deaths to that in TgTERT mice under a control diet, suggesting that TERT transgenic expression is partially the beneficial effects of CR.

By using longitudinal telomere length studies, we also describe here that CR delays telomere shortening associated to aging in blood cells from WT mice (PBLs), to an analogous degree to that observed associated to TERT over-expression. Other tissues, such as lung, kidney, bone marrow and muscle, also presented longer telomeres in mice under CR compared to those under the control diet. In agreement with a protection from telomere shortening associated with aging, we also observed that CR protected from telomere-originated DNA damage and chromosomal aberrations. This included “multitelomeric signals”, which have been recently associated to increased telomere fragility owing to replication fork stalling at telomeres [48]. The fact that CR reduced the load of telomere fragility, may be suggestive of a reduced replicative stress associated to CR *in vivo*, in agreement with the lower cellular proliferation described for this condition [53], [54]. Alternatively, the observed telomere protection associated to CR could also be explained by the reduction of oxidative stress mediated by CR. Oxidative stress accelerates telomere loss, whereas antioxidants decelerate it [55].

Importantly, the slow-down or even increase in telomere length associated to CR in mice, together with the protection from telomere damage, may underlie the delayed onset of age-related diseases upon CR. In support of this notion, there is recent evidence indicating that the rates of accumulation of short telomeres with aging determines mouse longevity [44]. Furthermore, short telomeres are associated with age-related diseases, including heart disease, impaired glucose tolerance, type 2 diabetes and higher plasma oxidative stress [56].

Interestingly, the significantly improved health-span associated to CR in WT mice, also lead to a delay in the onset of first deaths in the cohort, although was not sufficient to significantly increase life-span compared to control mice. Life-span extension in this setting was only achieved in TgTERT over-expressing mice, suggesting a synergy between high TERT expression and CR in extending mouse longevity. The fact that we did not observe a significant increase in the longevity in CR WT mice maybe related to the young age of mice used for this study (3 month of age) compared to other studies (>12 month of age), suggesting the interesting idea that CR may differentially impact on young or adult individuals, in line with the higher proliferative demands of younger organisms.

Finally, our results show a clear impact of CR reducing the tumor incidence associated to the TgTERT genotype. In particular, although TERT overexpressing mice presented a higher incidence of neoplasias than WT mice, the incidence of these neoplasias was reduced to a similar incidence than that of WT mice under CR. This results in a synergistic impact of both mechanisms on survival, mimicking the expression of tumor suppressors under a telomerase overexpression background [25].

In conclusion, we provide evidence of the interaction between two major anti-aging mechanisms in mammals. Further investigation of the interface between telomerase and CR could help unveiling how CR works and could permit to draw new and safer anti-aging treatments.

## Materials and Methods

### Ethics Statement

Male mice of a 100% C57BL/6 background were produced and stored at a pathogen-free barrier area of the CNIO, in accordance to the recommendations of the Federation of European Laboratory Animal Science Associations. Mice were inspected in a daily basis by an authorized animal facility expert and weight monthly the firsts 6 months and each two months thereafter. All the work carried out by the Animal Facility of CNIO (including mice welfare) complies with both national and EU legislation – Spanish Royal Decree RD 1201/2005 and EU Directive 86/609/CEE as modified by 2003/65/CE – for the protection of animals used for research experimentation and other scientific purposes. Mice were sacrificed using carbon dioxide (CO2) following ISCIII IACUC guidelines, when presenting signs of illness or tumors in accordance to the Guidelines for Humane Endpoints for Animals Used in Biomedical Research [57]. The date of euthanasia was considered as an estimation of the natural life span. The Spanish National Cancer Research Centre (CNIO) is part of the “Carlos III” Health Institute (ISCIII) and all protocols were previously subjected and approved by the Ethical Committee of the ISCIII; approval ID numbers: PA-418/08.

### Experimental Set-up

After weaning, five mice were housed per cage and fed *ad libitum* of a non-purified diet (n° 2018, Harlan). For the aging study, three-month old mice were individually housed and randomly assigned to control and CR group of 18–20 mice each group (WT = 20, TgTERT = 18, WT CR = 19, TgTERT CR = 19). Independently of specific cases delineated at the corresponding figure legends, two mice of each cohort were sacrificed after the 25 months’ time point for experimentation and were censored from the survival curves. Control mice were fed 92.5 kcal per week of chemically defined control diet (AIN-93M, Diet No. F05312, bioderv, Frenchtown, NJ). This caloric intake was found to be ∼10% fewer calories than average daily intake of C57BL/6 mice to ensure that all was consumed and allowing the comparison of non-obese controls and CR mice. Caloric restriction was introduced by feeding 74 kcal per week of chemically defined CR diet for two weeks, followed by 59.2 kcal per week of CR diet (AIN-93M 40% Restricted, Diet No. F05314, Bioserv). Defined diets were cold packed into 1 g pellets. Mice were fed two-seventh of the weekly allotment of food on Monday and Wednesdays and three-seventh of that amount of Friday [10], [58].

### Histological Analysis

Histopathology was performed as described [59]. Briefly, tissues and organs from sacrificed or natural death mice were fixed for 24 h in a 10% neutral-buffered formalin solution at room temperature, dehydrated through graded alcohols and xylene, and embedded in paraffin. Histological analysis was achieved on 4–5 µm sections according to standard procedures.

### Bone Density and Fat Content

Bone mineral density and fat content were measured in anesthetized animals (or post-mortem when referred) using a Dual Energy X-ray Absorptiometry (DEXA) scan device.

### Intraperitoneal Glucose Tolerance Tests

Mice were fasted for at least 8 hr, and injected intraperitoneally with a 50% dextrose solution (2 g/kg body weight) as previously described. Blood glucose levels were measured at the indicated time points with a glucometer (Glucocard Memory 2, Arkray, Japan), after injection. Analysis of the data was performed as described in [25].

### Insulin, HOMA-IR, IGF1 and GH Measurements

Blood insulin levels were measured with an Ultra-Sensitive Mouse Insulin ELISA Kit (Crystal Chem Inc. #90080), following manufacturers protocol. HOMA-IR was calculated as previously described [31]. IGF-1 levels were calculated with a Rat/Mouse IGF-1 ELISA (Novozymes, AC-18F1) and growth hormone with a Rat/Mouse ELISA Kit (Millipore, EZRMGH-45K) following manufactures proceedings. Mice were fasted for at least 6 hr prior to blood insulin, IGF-1 and GH analysis.

### Telomere QFISH on Paraffin Sections

Paraffin-embedded tissue sections were hybridized with a PNA-telomeric probe, and fluorescence intensity of telomeres was determined as previously described [59]. Quantitative image analysis was performed using the Definiens Developer Cell software (version XD 1.2; Definiens AG). For statistical analysis a two-tailed Student *t*-test was used to assess significance (GraphPad Prism software).

### Quantitative Telomere Fluorescence *in situ* Hybridization (QFISH) on Bone Marrow Cells

Bone marrow cells were isolated and stimulated with IL3 (10 ng/µl) (Sigma) diluted in 25% RPMI (Sigma) 75% Myelocult (Stemcell technologies) culture medias for 72 hr. Cells were incubated with 0.1 mg/mL Colcemid (Gibco) at 37°C overnight to arrest cells in metaphase. Cells were then harvested and fixed in methanol:acetic acid (3:1). Metaphases were spread on glass slides and dried overnight, and QFISH was performed using a PNA-telomeric probe labeled with Cy3 as described previously [29], [60], [61]. Images were captured at 100× magnification using a COHU CCD camera on a Leica DMRA (Leica, Heidelberg, Germany) microscope. Telomere fluorescence signals were integrated from 17–21 metaphases from 2 animals per diet group and genotyped and quantified by using the TFL-TELO program (gift from Peter Lansdorp, Vancouver, Canada) [60]. Telomere fluorescence values were converted into kb by external calibration with the L5178Y-S and L5178Y-R lymphocyte cell lines with known telomere lengths of 10.2 and 79.7 kb, respectively [61].

### High-througput QFISH (HT QFISH) on Peripheral Blood Leukocytes

Blood was extracted from the facial vein while the mice were alive at the indicated time points from the beginning of the diet. Erythrocyte lysis was performed (Buffer EL, Quiagen) and peripheral blood leukocytes were plated on a clear bottom black-walled 96-well plate. HT-QFISH was performed as previously described [28]. Telomere length values were analyzed using individual telomere spots (>5000 per sample) corresponding to the specific binding of a Cy3 labeled telomeric probe. Fluorescence intensities were converted into Kb as previously described [28], [61].

### Quantification of Phosphorylated H2AX

We performed γ-H2AX (Millipore 05-636, clone JBW301) staining on paraformaldehyde-fixed, paraffin-embedded sections (AxioVision software was used for image analysis; quantitation of immunostainings was determined by counting the number of peroxidase stained nuclei over the total number of hematoxylin-stained nuclei). Samples were processed and acquired under identical conditions.

### Neuromuscular Coordination Test

The tightrope test was performed as described elsewhere [25].

## Supporting Information

Figure S1 **Molecular markers of aging in WT and TgTERT mice under Control and CR diets.** (A and B) Femur bone mineral density (BMD) was measured at 16 months of diet (A) and 24 months of diet (B) in mice from the different cohorts. Values are given as average ± SEM, and statistical significance was determined by the two-tailed Student’s t-test. (C) Femur bone mineral density (BMD) variation through lifetime of WT and TgTERT mice under control and CR diets. Values are given as average. (D and E) IGF-1 (D) and GH (E) in serum were measured at 16 months of diet in mice of the indicated cohorts. The number of mice is indicated on the top of each bar (n). Values are given as average ± SEM, and statistical significance was determined by the two-tailed Student’s *t*-test.
(TIF)

Figure S2 **Protection from DNA damage in mice under calorie restriction.** (A) Percentage of γ-H2AX positive cells in the kidney of mice from the indicated cohorts. Student´s t-test was used for statistical assessments. (B) Representative γ-H2AX immunohistochemistry images of kidney from the indicated mice cohorts.
(TIF)

Figure S3 **Slower age-dependent telomere shortening in mice under calorie restriction.** (A, B and C) Percentage of short telomeres (<2 kb, <5 kb and <10 kb; A, B and C respectively) was determined by HT QFISH on white blood cells from the indicated mice under CR or control diet at different time points. The number of mice is indicated on the top of each bar (n). Values are given as average ± SEM, and statistical significance was determined by one-tailed Student’s *t*-test.
(TIF)

Figure S4 **Calorie restriction leads to telomere maintenance and/or elongation with time in a percentage of mice.** (A, B and C) The behavior of mean telomere length was classified in three different profiles (“Decrease”, “Increase”, and “Maintenance”) at different times of diet (1–22 months of diet, 5–22 months of diet and 9–22 months of diet; A, B and C, respectively) in the indicated groups. Numbers above bars indicate the number of mice showing the profile of interest over the total number of mice. Chi-squared test was used to assess the statistical significance of the differences observed. (D, E and F) The behavior in the percentage of short telomeres (<15 kb) was classified in three different profiles (“Increase”, “Decrease”, and “Maintenance”) at different times of diet (1–22 months of diet, 5–22 months of diet and 9–22 months of diet; D, E and F respectively) in the indicated groups. Numbers above bars indicate the number of mice with the profile of interest over the total number of mice. Chi-squared test was used to assess the statistical significance of the differences observed.
(TIF)
