# Supplementary figures and images for: Telomerase Reverse Transcriptase Synergizes with Calorie Restriction to Increase Health Span and Extend Mouse Longevity
Source: PLoS One. 2013 Jan 22;8(1):e53760. doi: 10.1371/journal.pone.0053760 (PMC3551964; doi:10.1371/journal.pone.0053760)

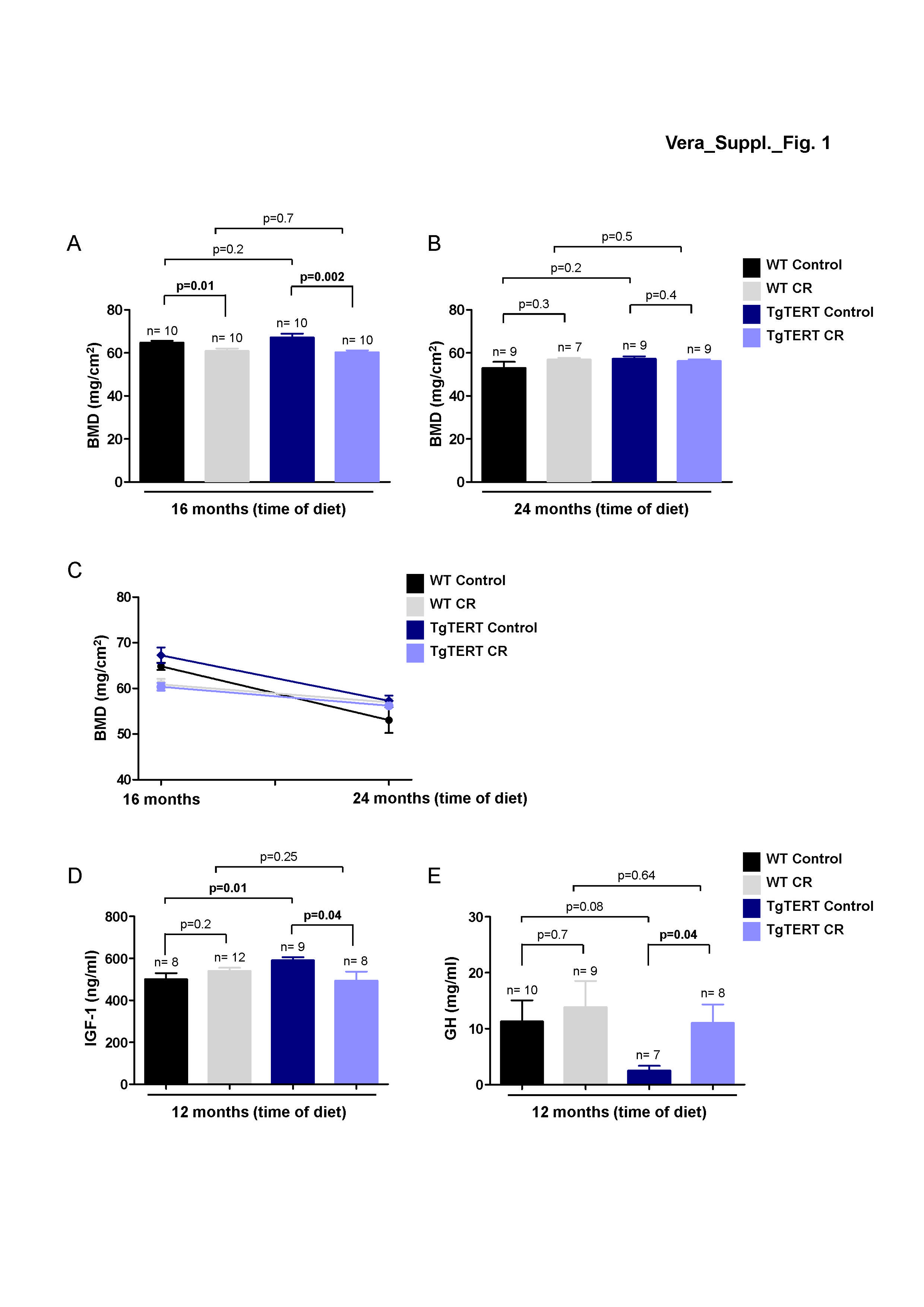

Supplement: Figure S1 — Molecular markers of aging in WT and TgTERT mice under Control and CR diets. (A and B) Femur bone mineral density (BMD) was measured at 16 months of diet (A) and 24 months of diet (B) in mice from the different cohorts. Values are given as average ± SEM, and statistical significance was determined by the two-tailed Student’s t-test. (C) Femur bone mineral density (BMD) variation through lifetime of WT and TgTERT mice under control and CR diets. Values are given as average. (D and E) IGF-1 (D) and GH (E) in serum were measured at 16 months of diet in mice of the indicated cohorts. The number of mice is indicated on the top of each bar (n). Values are given as average ± SEM, and statistical significance was determined by the two-tailed Student’s t-test. (TIF) [file pone.0053760.s001.tif]

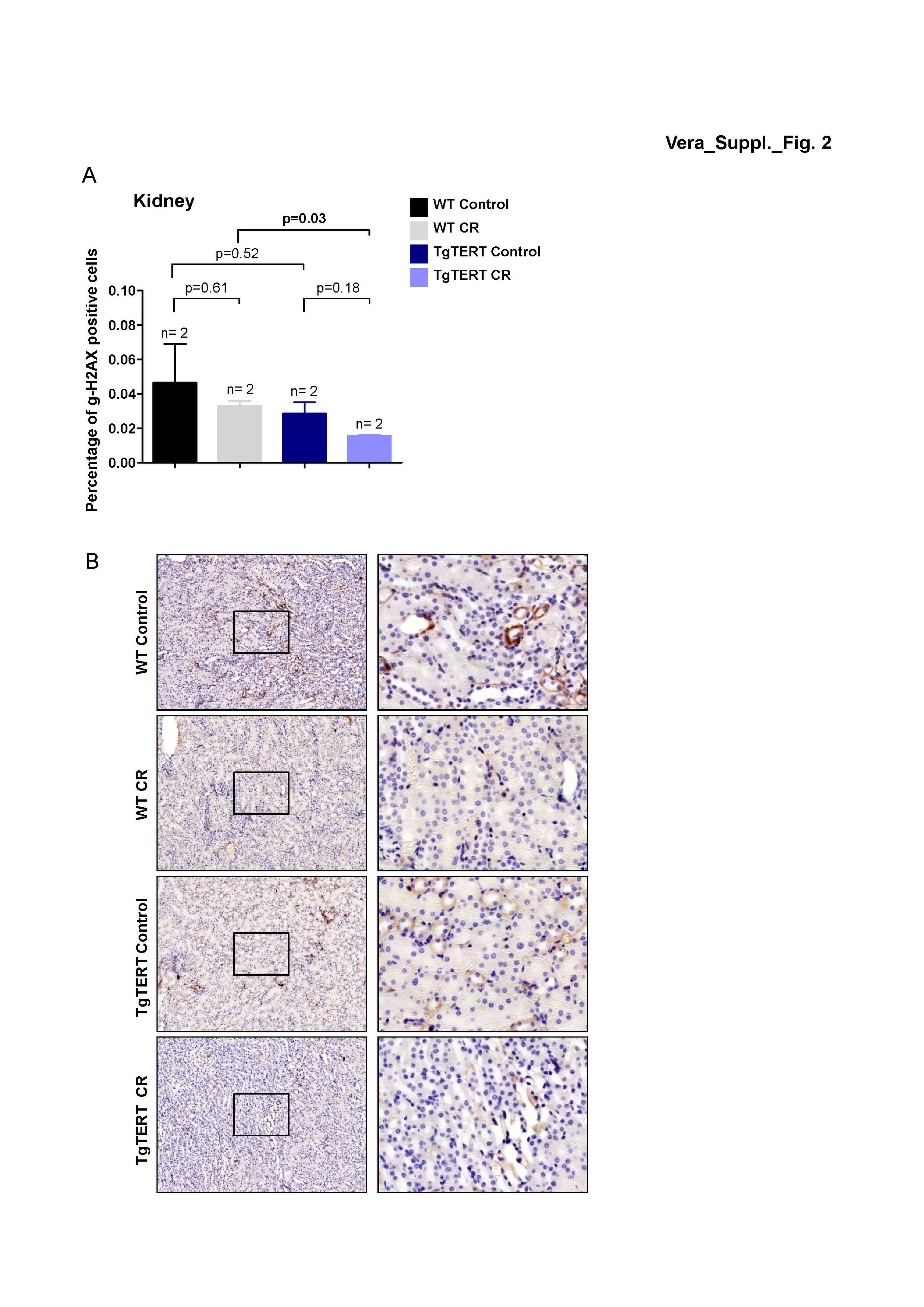

Supplement: Figure S2 — Protection from DNA damage in mice under calorie restriction. (A) Percentage of γ-H2AX positive cells in the kidney of mice from the indicated cohorts. Student´s t-test was used for statistical assessments. (B) Representative γ-H2AX immunohistochemistry images of kidney from the indicated mice cohorts. (TIF) [file pone.0053760.s002.tif]

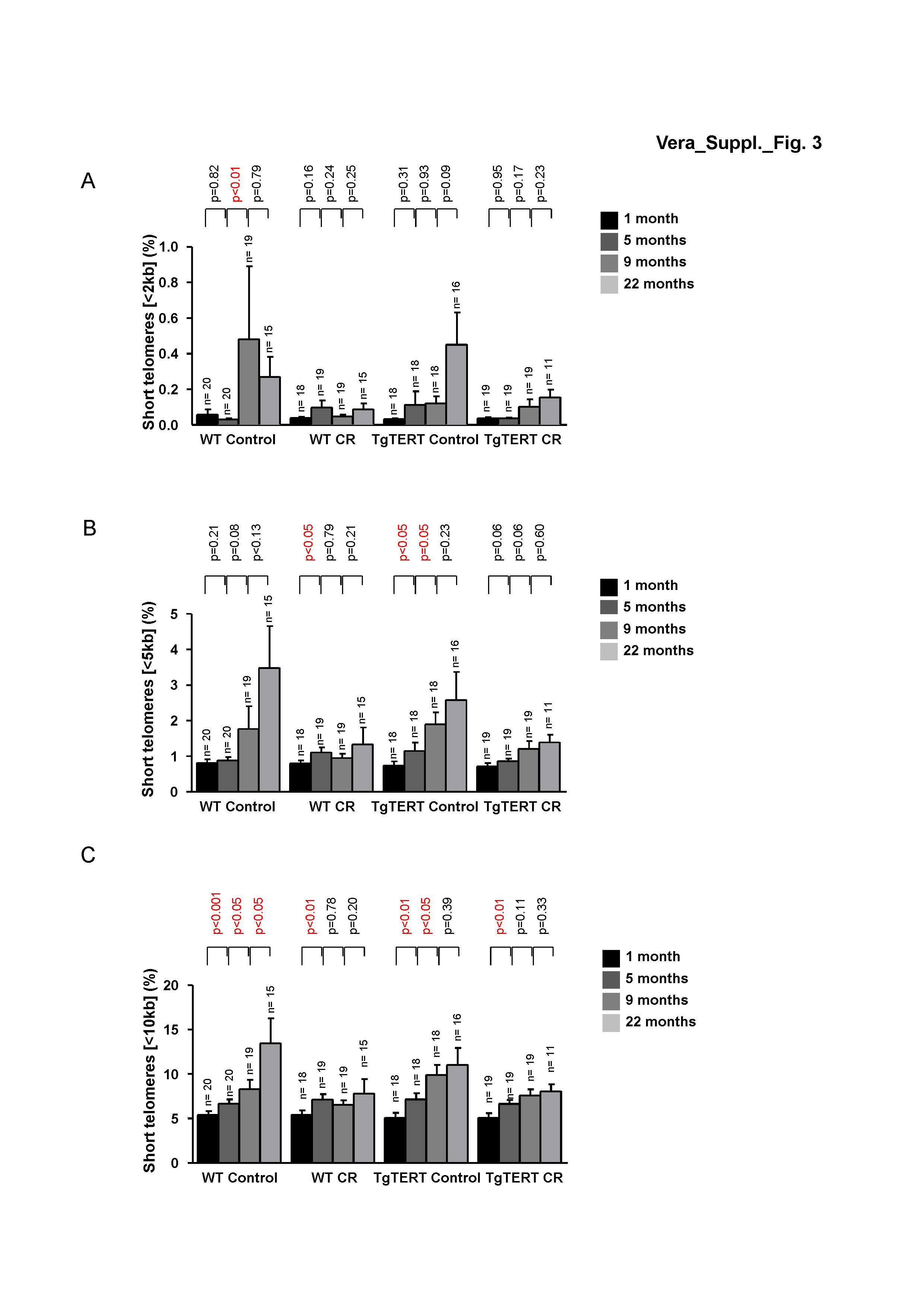

Supplement: Figure S3 — Slower age-dependent telomere shortening in mice under calorie restriction. (A, B and C) Percentage of short telomeres (<2 kb, <5 kb and <10 kb; A, B and C respectively) was determined by HT QFISH on white blood cells from the indicated mice under CR or control diet at different time points. The number of mice is indicated on the top of each bar (n). Values are given as average ± SEM, and statistical significance was determined by one-tailed Student’s t-test. (TIF) [file pone.0053760.s003.tif]

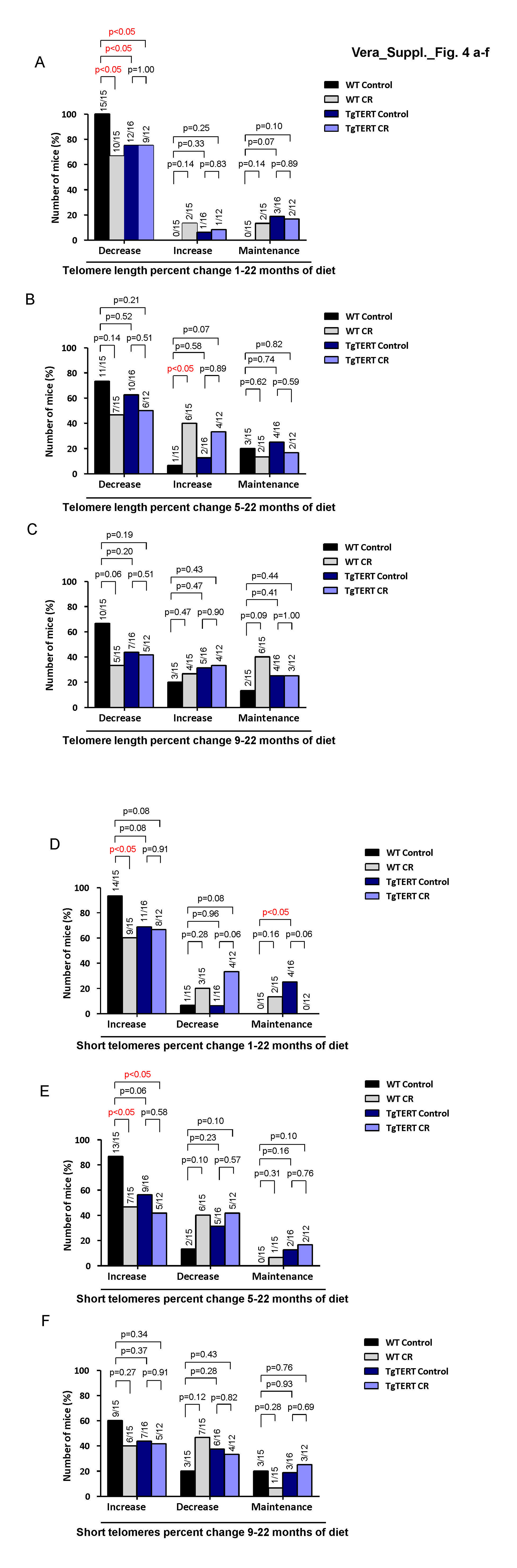

Supplement: Figure S4 — Calorie restriction leads to telomere maintenance and/or elongation with time in a percentage of mice. (A, B and C) The behavior of mean telomere length was classified in three different profiles (“Decrease”, “Increase”, and “Maintenance”) at different times of diet (1–22 months of diet, 5–22 months of diet and 9–22 months of diet; A, B and C, respectively) in the indicated groups. Numbers above bars indicate the number of mice showing the profile of interest over the total number of mice. Chi-squared test was used to assess the statistical significance of the differences observed. (D, E and F) The behavior in the percentage of short telomeres (<15 kb) was classified in three different profiles (“Increase”, “Decrease”, and “Maintenance”) at different times of diet (1–22 months of diet, 5–22 months of diet and 9–22 months of diet; D, E and F respectively) in the indicated groups. Numbers above bars indicate the number of mice with the profile of interest over the total number of mice. Chi-squared test was used to assess the statistical significance of the differences observed. (TIF) [file pone.0053760.s004.tif]
